# Deceased serum bilirubin and albumin levels in the assessment of severity and mortality in patients with acute pancreatitis

**DOI:** 10.7150/ijms.49606

**Published:** 2020-09-23

**Authors:** Xiao Xu, Fen Ai, Min Huang

**Affiliations:** 1 Department of Emergency, The Central Hospital of Wuhan, Tongji Medical College, Huazhong University of Science and Technology, Wuhan Hubei, 430014, China; 2 Department of Nephrology, The Central Hospital of Wuhan, Tongji Medical College of Huazhong University of Science and Technology, Wuhan Hubei, 430014, China

**Keywords:** Acute pancreatitis, Total bilirubin, Serum albumin, Nomogram, Mortality

## Abstract

**Background:** Our study investigated the diagnostic and prognostic role of serum antioxidant indexes in patients with acute pancreatitis (AP).

**Methods:** This study included 708 AP patients from the Medical Information Mart for Intensive Care-III (MIMIC-III) database and 477 patients from the eICU Collaborative Research Database (eICU-CRD). X-tile software was applied to determine the best cutoff values for serum antioxidant indexes. Univariate and multivariate regression analyses were employed to select variables associated with severe AP (SAP) and in-hospital mortality. Finally, the nomograms were also externally validated in the eICU-CRD.

**Results:** The best cutoff values for serum total bilirubin (TBIL) and albumin were 1.1 mg/dL and 2.1 g/dL in the training set, respectively. Multivariate logistical regression indicated that both TBIL (odds ratio [OR]=0.740, 95% confidence interval [CI]: 0.616-0.889, P=0.001) and albumin (OR=0.890, 95%CI: 0.819-0.967, P=0.006) were independent risk factors for SAP. Similarly, multivariate Cox analysis revealed that serum TBIL (hazard ratio [HR]=0.768, 95%CI:0.635-0.928, P=0.006) and albumin (HR=0.962, 95%CI:0.927-0.998, P=0.037) were independent risk factors for in-hospital mortality in AP patients. The diagnostic nomogram containing TBIL, albumin, Sequential Organ Failure Assessment (SOFA) score and urea nitrogen and prognostic nomogram combining TBIL, albumin, white blood count, SOFA score, and age obtained good discrimination, calibration and clinical utility in both the MIMIC-III and eICU-CRD.

**Conclusion:** Serum TBIL and albumin were independent predictors for SAP and in-hospital mortality in AP patients. The nomograms combining serum TBIL and albumin with other significant features exerted favorable predictive performance for SAP and in-hospital mortality.

## Introduction

Acute pancreatitis (AP) is a sudden inflammatory disease involved in the pancreas that is associated with substantial morbidity^1^. The global incidence of AP is 34 per 100,000 individuals^2^, with an increasing number of hospitalizations every year worldwide^3^. AP is characterized by local and systemic inflammation ranging from mild localized disease to severe systemic inflammatory disease^4^. AP is associated with significant mortality: up to 30% in patients with severe AP (SAP) due to multiple organ failure^5^. Hence, it is clinically significant to identify novel biomarkers to predict AP severity and mortality with high accuracy.

Oxidative stress is a common pathway mediated by reactive oxygen species (ROS), DNA oxidation, lipid peroxidation, and protein oxidation^6^. Excessive oxidative stress is implicated in the injury of acinar cells, as observed in a murine model of AP^7^, ^8^. Moreover, clinical evidence has also shown that oxidative stress is common in the early phase in patients with AP^9^. More importantly, several studies showed that antioxidant agents were effective in reducing acinar cell necrosis and decreasing the severity of pancreatic tissue injury in a murine model of AP^10^, ^11^. These previous findings confirmed the essential role of oxidative stress in AP induction and progression in both human and murine models.

Serum bilirubin has long been viewed as the end product of heme catabolism. However, as an endogenous antioxidant under physiological conditions, it has recently been reported to play an important part in inhibiting oxidative stress^12^, ^13^. Bilirubin is known as a potent antioxidant which could suppress oxidative stress more powerfully than other antioxidant agents, such as superoxide dismutase (SOD) or vitamin E^14^. The strong antioxidant effects of bilirubin are due to its rapid regeneration by biliverdin reductase after being oxidized to biliverdin^14^. Moreover, the antioxidant value of albumin has attracted attention of medical scientists due to its free radical-trapping properties, which could decrease oxidative stress by binding ROS^15^. Although the associations between oxidative stress and bilirubin as well as albumin have been illustrated in diseases including chronic obstructive pulmonary disease^16^, ^17^, cardiovascular disease^18^, ulcerative colitis^19^, rheumatoid arthritis^20^, and cancer^21^, no study has specifically assessed the association between serum bilirubin as well as albumin and AP with regard to oxidative stress.

Thus, the present study, initially investigated whether serum bilirubin and albumin concentrations were associated with the disease severity of AP. We then evaluated the predictive value of a survival nomogram based on serum bilirubin, albumin, and other significant features in the Medical Information Mart for Intensive Care-III (MIMIC-III) database. Finally, we further validated the diagnostic and prognostic significance of the nomograms in 499 AP patients from an independent database, the eICU Collaborative Research Database (eICU-CRD).

## Method

### Data source

The data in the training set was extracted from a large critical care database, MIMIC-III database. The MIMIC-III is a public database freely available to all medical users^22^. We also analyzed another critical care database, the eICU-CRD^23^ to verify the conclusions drawn from the MIMIC-III. The study was consistent with the statement of the Transparent Reporting of a multivariable prediction model for Individual Prognosis Or Diagnosis (TRIPOD)^24^. As this study was an analysis of public databases, it was exempt from the requirement for informed consent from patients and approval of the Institutional Review Board (IRB). After finishing the web-based training courses and the Protecting Human Research Participants examination (No.8452818), we obtained permission to extract data from the MIMIC III and eICU-CRD.

### Study population

**Figure 1:**
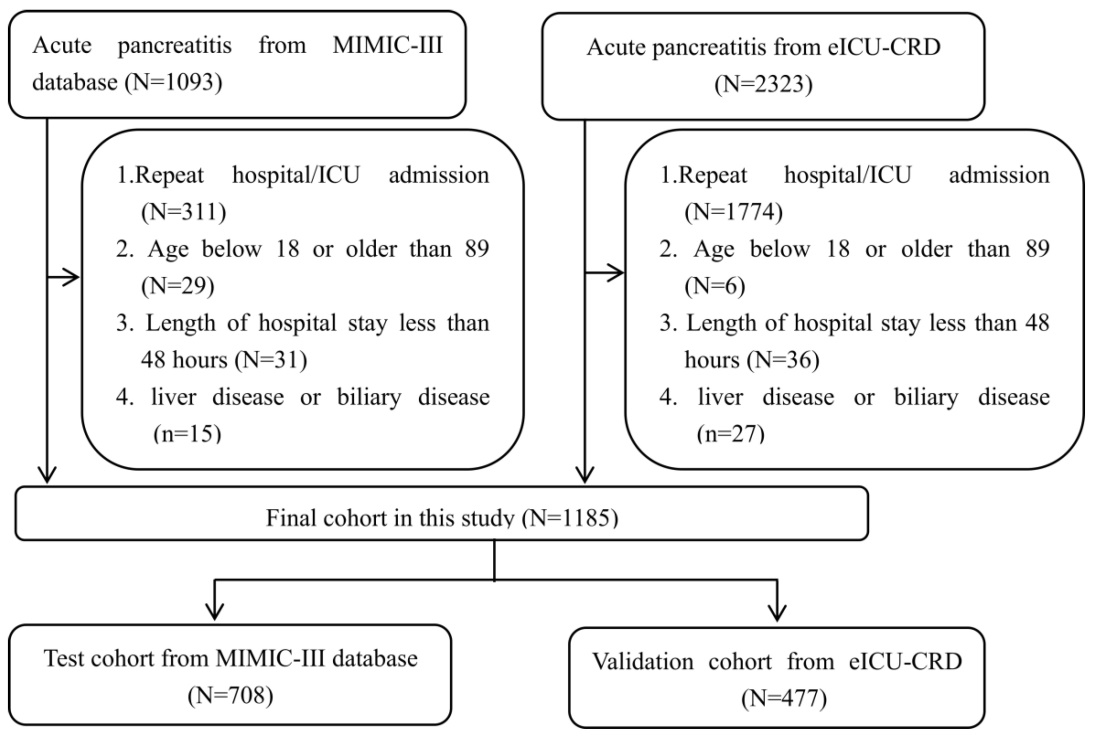
Flow chart of patient selection.

The analyses included patients with confirmed AP. The AP severity was classified according to the 2012 Atlanta Classification Criteria^25^ as mild, moderate, or severe). Patients with (1) repeat hospital/intensive care unit (ICU) admission; (2) age below 18 or older than 89 years; (3) length of hospital stay of fewer than 48 hours; and (4) liver or biliary disease that could affect the bilirubin or albumin levels were excluded from this study. Thus, this study finally included 708 and 477 patients with AP in the training and validation sets, respectively. The detailed process of selection is shown in Figure 1.

### Data collection

Demographic features collected on admission, such as age, gender, race and body mass index (BMI) were extracted from MIMIC-III database. Additionally, comorbidities (Chronic kidney disease, hypertension, diabetes and chronic heart failure), complications (acute kidney injury, sepsis), therapeutic and clinical management (vasopressor usage, mechanical ventilation, renal replacement therapy, endoscopic necrectomy, surgical necrectomy, endoscopic retrograde cholangiopancreatography) were also included in this analysis. More importantly, biochemical indexes on admission were collected. We simplified the severity of AP by combining mild AP with moderate AP as non-severe acute pancreatitis (non-SAP). As the eICU-CRD was used as a validation dataset, so we extracted variables as identical as possible within the variables from MIMIC-III database.

### Outcome assessment

The primary goal of our study was to evaluate the predictive value of the diagnostic nomogram including oxidative stress indexes for SAP. The secondary objective of this study was to assess the predictive significance of the survival nomogram for in-hospital mortality in patients with AP.

### Statistical analysis

Data analyses were completed using IBM SPSS Statistics for Windows version 22.0 and R software version 3.3.1. X-tile software provides an accurate assessment of every possible method of dividing the population into low-level and high-level marker expression, and provides an easy-to-use solution of cut-point selection^26^. The clinical features between high total bilirubin (TBIL) and low TBIL groups were then analyzed with Student's t- or chi-square tests, as appropriate. Mann-Whitney tests were adopted to compare differences in continuous parameters with skewed distributions. Moreover, a logistic regression model was utilized to identify significant variables predictive of SAP and Cox regression analysis was performed to identify informative indexes significantly associated with mortality in the MIMIC-III database. Finally, the nomogram was created based on the selected variables from the MIMIC-III database and further validated with data from the eICU-CRD. P-values smaller than 0.05 at both tails were considered statistically significant.

## Results

### Baseline data from the MIMIC-III database

A total of 1,093 patients with AP from the MIMIC-III database were initially screened for eligibility, of which 708 met the inclusion criteria and were finally enrolled in the retrospective cohort. Among these, 206 and 502 patients were identified as SAP and non-SAP according to the 2012 Atlanta Classification Criteria. The included patients with AP were divided into high and low TBIL groups according to the optimal cutoff value of TBIL (1.1 mg/dL). As shown in Table 1, sepsis (P<0.001), acute kidney disease (P<0.001), vasopressor usage (P<0.001), rate of mechanical ventilation (P<0.001), renal replacement therapy (P<0.001), endoscopic retrograde cholangiopancreatography (ERCP) (P<0.001), Sequential Organ Failure Assessment (SOFA) score (P<0.001), Outcome and Assessment Information Set (OASIS) score (P<0.001), hemoglobin (P<0.0001), urea nitrogen (P<0.0001), serum creatinine (P<0.001), alkaline phosphatase (P=0.031), SAP (P<0.001), and in-hospital mortality (P<0.001) differed significantly between the two groups.

### Prognostic significance of TBIL, direct bilirubin (DBIL), and albumin levels

**Figure 2:**
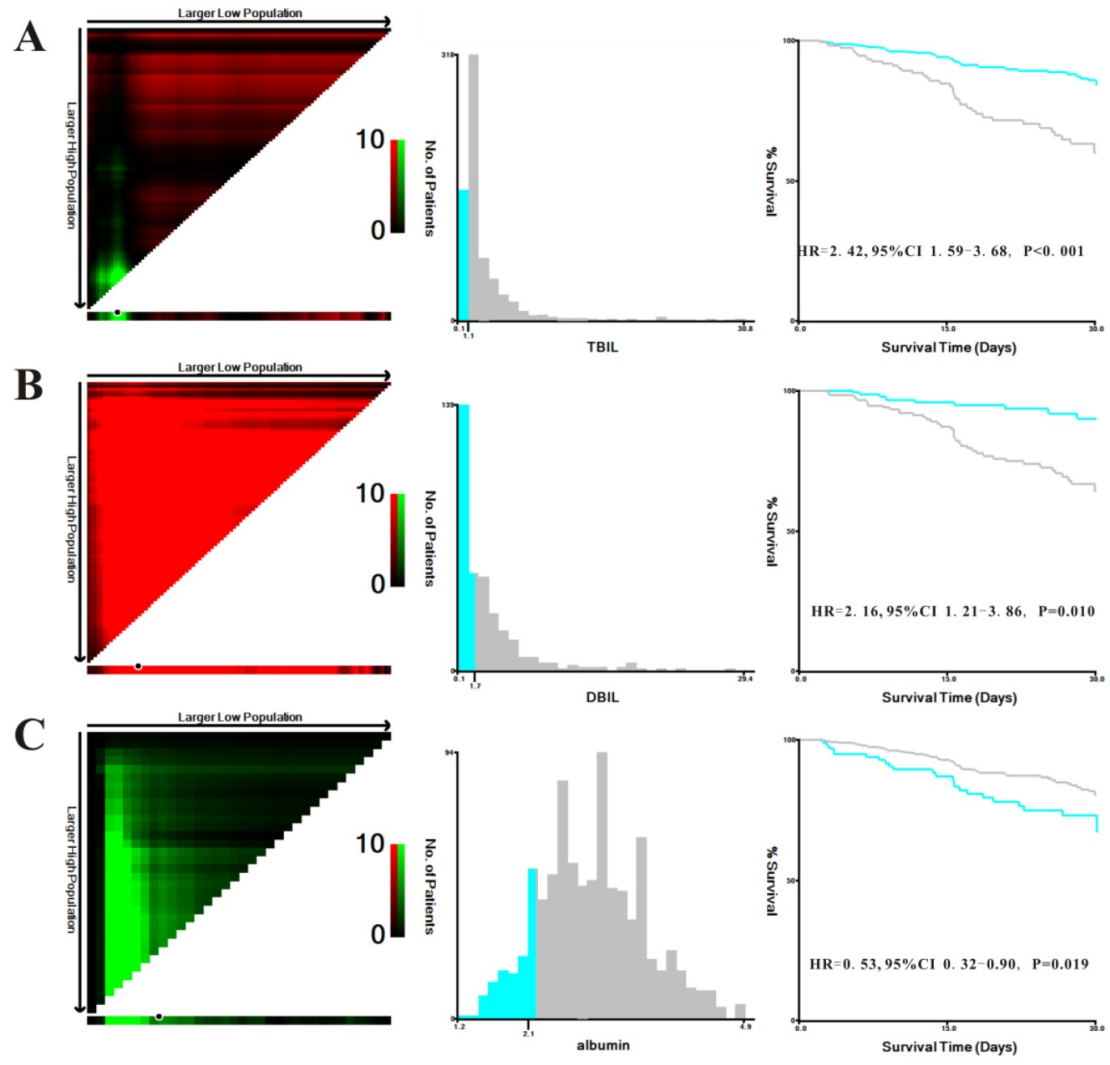
X-tile analyses of total bilirubin (TBIL) (**A**), direct bilirubin (DBIL) (**B**), and albumin (**C**) concentrations in the Medical Information Mart for Intensive Care-III (MIMIC-III) database. X-tile plots for patients with acute pancreatitis are shown on the left panels; the black circles indicate the optimal cutoff values, which are also presented in histograms (middle panels). Kaplan-Meier curves are shown in the right panels.

We explored the prognostic significance of TBIL, DBIL and albumin in patients with AP. X-tile was used to determine the optimal cutoff values of 1.1 mg/dL for TBIL, 1.7 mg/dL for DBIL, and 2.1 g/dL for albumin based on in-hospital mortality in MIMIC-III patients (Figure 2). In addition, the Kaplan-Meier plots demonstrated that higher levels of TBIL (hazard ratio [HR]=0.414, 95% confidence interval [CI]: 0.272-0.631, P<0.001), DBIL (HR=0.464, 95%CI:0.259-0.829, P=0.010), and albumin (HR=0.534, 95%CI: 0.315-0.904, P=0.019) were associated with better survival in patients with AP.

### A predictive nomogram for SAP

**Figure 3:**
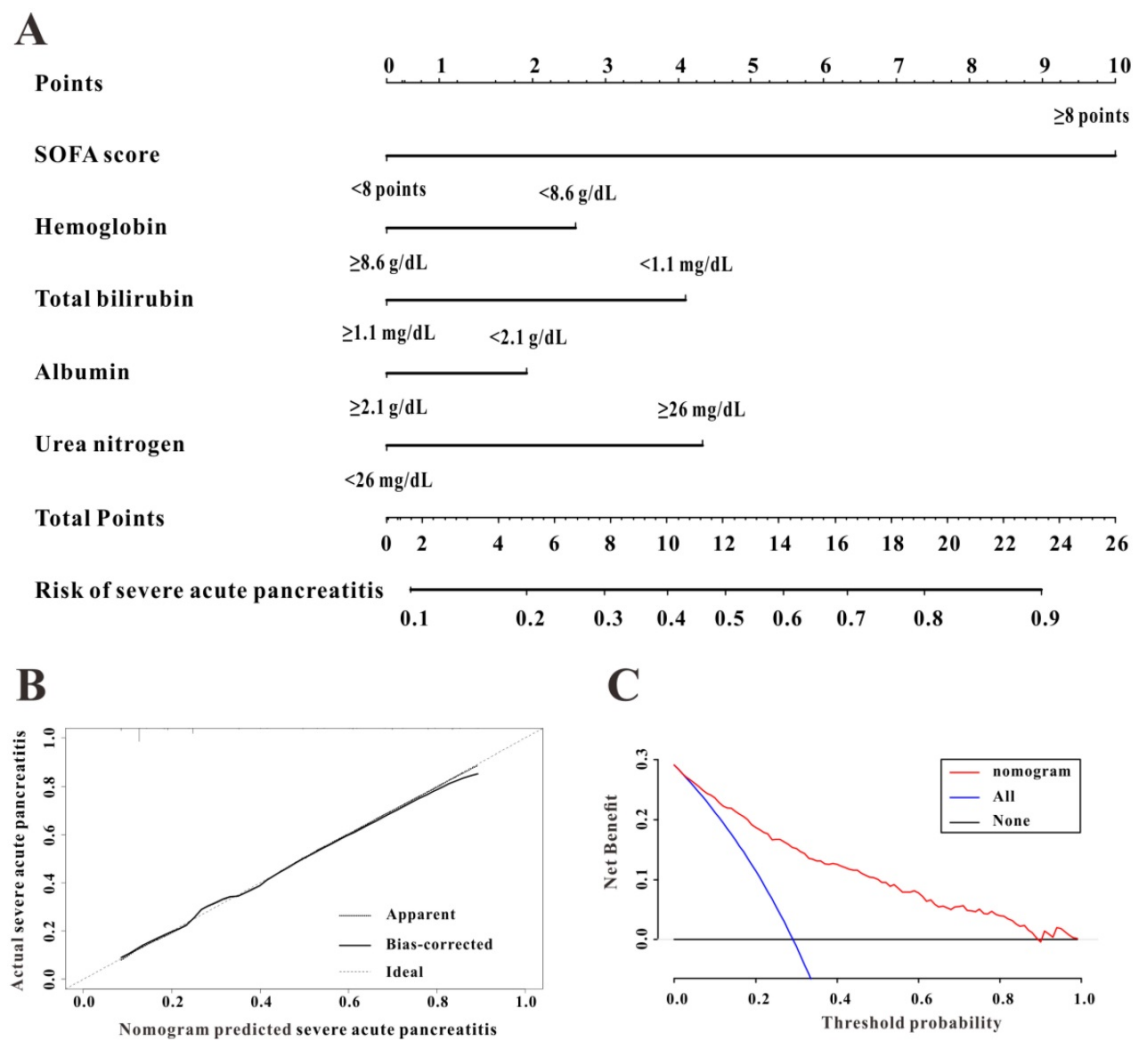
The discrimination, calibration, and clinical utility of the diagnostic nomogram. The diagnostic nomogram (**A**), calibration curve (**B**), and decision curves analysis (**C**) for predicting the risk for severe acute pancreatitis in the Medical Information Mart for Intensive Care-III (MIMIC-III) database are shown.

To construct a predictive nomogram for SAP, logistical regression analyses were conducted to screen for informative variables associated with SAP. Multivariate regression (Table 2) revealed SOFA score (P<0.001), hemoglobin (P=0.001), serum albumin (P=0.006), TBIL (P=0.001), and urea nitrogen (P<0.001) as independent risk factors for SAP. We established a predictive nomogram based on these five indexes (Figure 3A); the predictive ability of the diagnostic nomogram as measured by C-index was 0.855 (95% CI: 0.824-0.885).

A calibration curve used to evaluate the calibration ability of the nomogram showed that the predicted risk values were consistent with the actual values, indicating the good calibration ability of the predictive nomogram (Figure 3B). As shown in Figure 3C, for a threshold probability over 0.3, the diagnostic nomogram for SAP showed more benefit than treating either all or no patients, implying that the nomogram was clinically useful for the prediction of SAP.

### A survival nomogram for in-hospital mortality

**Figure 4:**
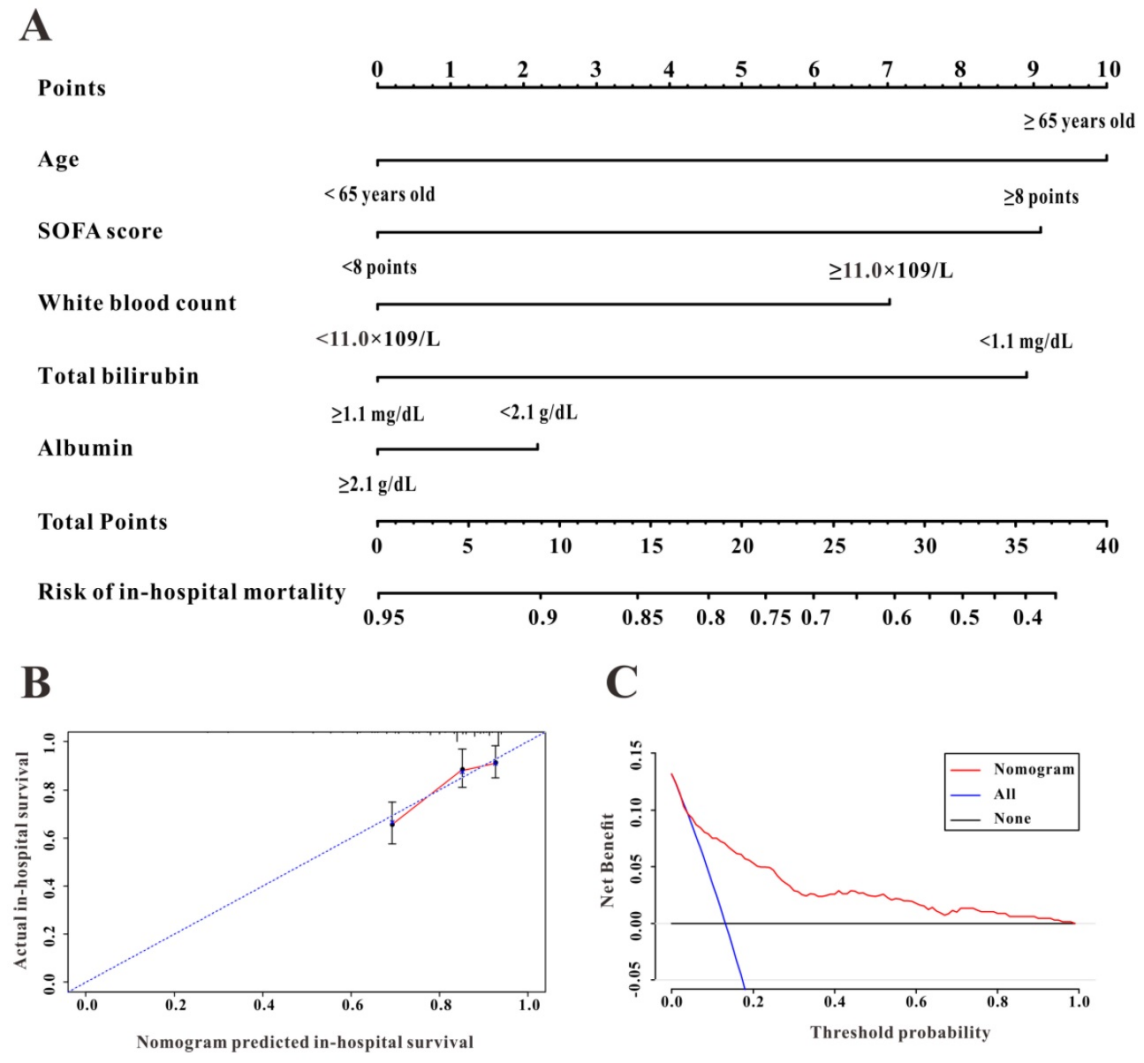
The discrimination, calibration, and clinical utility of the survival nomogram. The survival nomogram (**A**), calibration curve (**B**), and decision curves analysis (**C**) for predicting in-hospital mortality for patients with acute pancreatitis in the Medical Information Mart for Intensive Care-III (MIMIC-III) database are shown.

**Figure 5:**
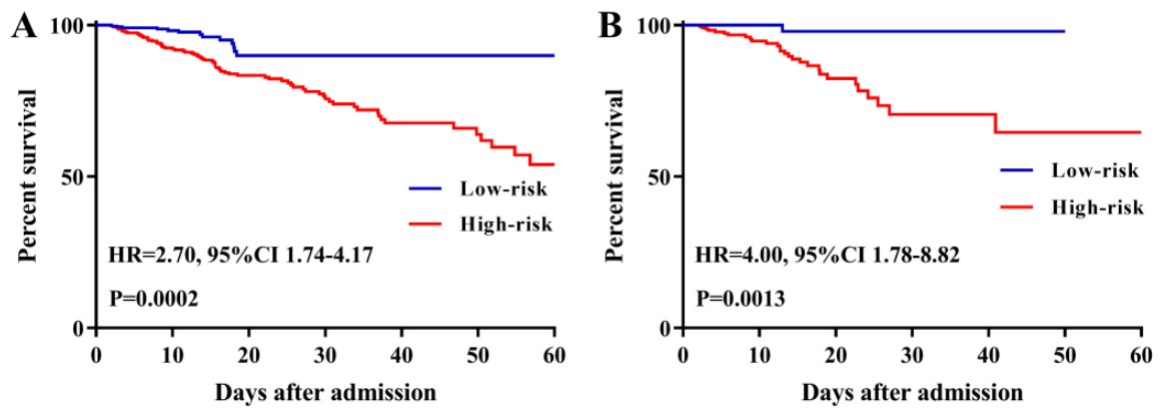
Kaplan-Meier curves of acute pancreatitis patients stratified by the median value of the survival nomogram in the Medical Information Mart for Intensive Care-III (MIMIC-III) (**A**) and eICU Collaborative Research Database (eICU-CRD) (**B**) databases.

To further evaluate the predictive ability of TBIL and albumin in patients with AP, we constructed a survival nomogram to predict in-hospital mortality based on the results of the multivariate analyses. Multivariate Cox regression (Table 3) revealed age (P<0.001), SOFA score (P<0.001), while blood count (P=0.002), serum albumin (P=0.037), and TBIL (P=0.006) as independent risk factors significantly associated with in-hospital mortality. The survival nomogram was constructed based on the final multivariate Cox model (Figure 4A); its predictive ability for in-hospital mortality as reflected by C-index was 0.821 (95% CI: 0.771-0.872). We then divided the AP patients into low- or high-risk groups according to the median value of the survival nomogram. The Kaplan-Meier curve showed better survival for AP patients in the low-risk group than that in the high-risk group (Figure 5A). In addition, the calibration curve of the survival nomogram showed similar predicted mortality rates to the actual observations (Figure 4B). The decision curve analysis (DCA) plot showed that, for a threshold probability over 1.5, the predictive nomogram for in-hospital mortality showed more benefit than treating either all or no patients, implying the clinical utility of the survival nomogram for the prediction of in-hospital mortality (Figure 4C).

### Validation in the eICU-CRD

**Figure 6:**
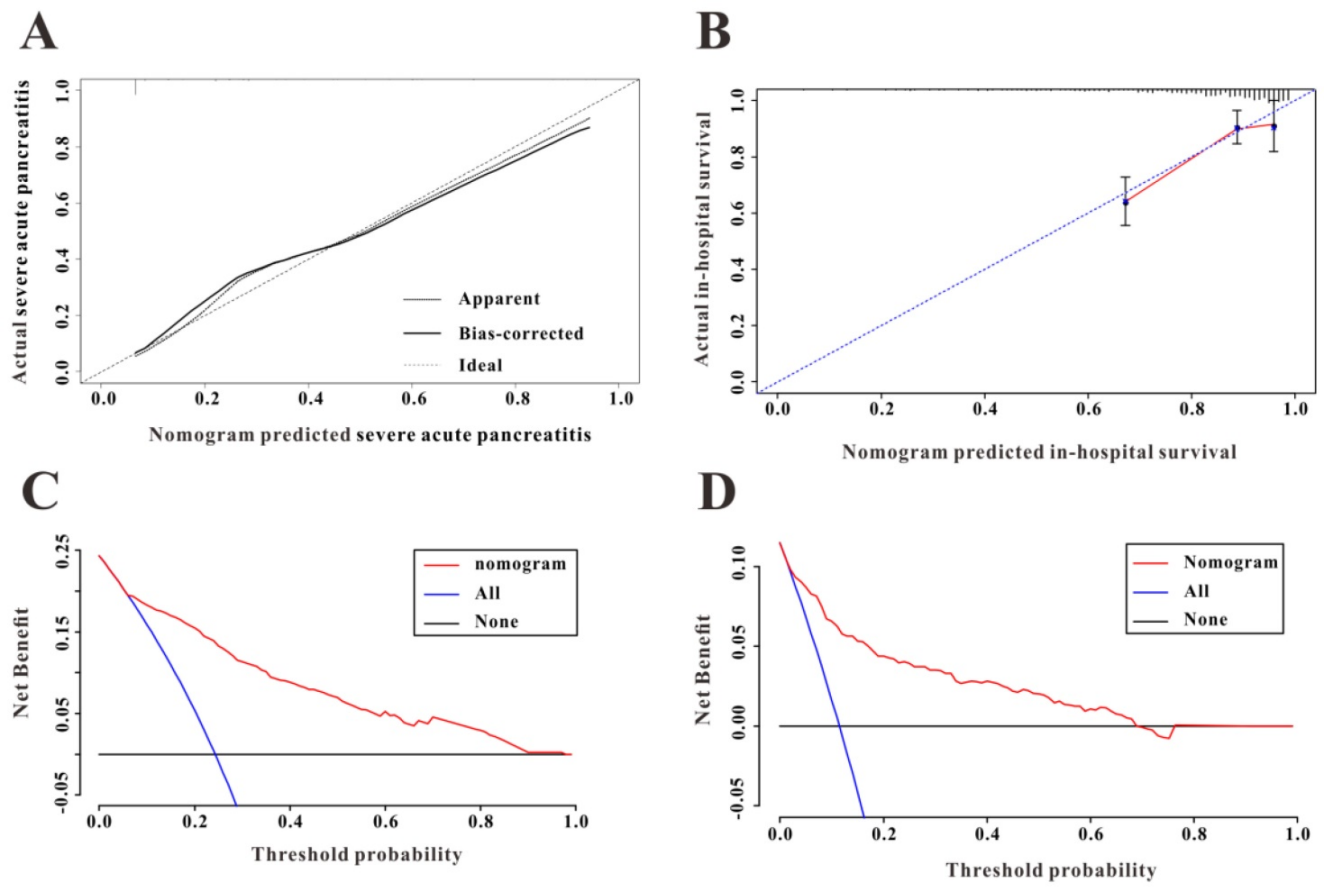
(**A**) The calibration curve for predicting severe acute pancreatitis in the eICU Collaborative Research Database (eICU-CRD). (**B**) The calibration curve for predicting in-hospital mortality in the eICU-CRD. (**C**) Decision curve analysis (DCA of the nomogram to predict severe acute pancreatitis in the eICU-CRD. (**D**) DCA of the nomogram to predict in-hospital mortality in the eICU-CRD.

Among 2,323 patients with AP screened for eligibility from the eICU-CRD, 408 were finally included in the present clinical study. The patients from eICU-CRD were used as a validation set to verify the results drawn from the MIMIC-III database. The results of the application of the predictive nomogram to these 408 AP patients indicated similar predictive performance, with a C-index of 0.879 (95%CI: 0.844-0.914). After stratifying the AP patients into low-risk or high-risk groups, the Kaplan-Meier curves showed longer survival time for patients in the low-risk group compared to that in the high-risk group (Figure 5B). Moreover, this nomogram also possessed good calibration ability (Figure 6A) and showed promising clinical utility for the prediction of SAP (Figure 6C). The survival nomogram showed good predictive accuracy (C-index: 0.822, 95%CI: 0.744-0.901) in validation based on 408 AP patients from the eICU-CRD. As shown by the calibration curve (Figure 6B), the survival nomogram slightly underestimated the predicted probabilities of in-hospital mortality compared to the actual observed values. The DCA plot also demonstrated the clinical usefulness of the survival nomogram in verification with data from eICU-CRD (Figure 6D).

### Comparison with SOFA and OASIS

As SOFA and OASIS are commonly used in the clinical setting to predict the severity and mortality in patients with AP, we compared the predictive significance of the nomograms with both instruments. As shown in Table 4, the nomograms combining serum TBIL and albumin with other significant variables showed a relatively high C-index compared to those for SOFA and OASIS in both the MIMIC-III and eICU-CRD, a difference that was statistically significant (De Long test). Therefore, the nomograms combining serum TBIL and albumin with other informative characteristics might have more predictive significance than any of these variables alone.

## Discussion

AP is the second-most common cause of total hospital stays and the fifth-leading disease of in-hospital mortality^27^. In the present study, we used two large ICU databases to explore the diagnostic and prognostic significance of serum TBIL and albumin in patients with AP. To our knowledge, this is the first clinical study to demonstrate that elevated serum TBIL and albumin levels were positive diagnostic factors for the prediction of SAP and independent prognostic factors for in-hospital mortality in patients with AP based on the results of multivariate regression analyses. Furthermore, both the diagnostic and survival nomograms including serum TBIL and albumin showed good performance for predicting outcomes. Likewise, the calibration curves for the two nomograms also showed good consistency between the predicted probabilities and the observational values. Encouragingly, validation data from the eICU-CRD showed consistent results.

Quite a few studies have reported that the pathogenesis of AP is closely related to oxidative stress^28^-^31^. Some studies have reported decreased antioxidant defense in patients with AP and that these patients are more vulnerable to damage from ROS^29^, ^32^, ^33^. ROS regulate the activation of the inflammatory response and the recruitment of inflammatory cells, eventually causing tissue damage in patients with AP^34^. Although the most direct indexes of oxidative stress are SOD, glutathione (GSH), malondialdehyde (MDA), and glutathione peroxidase (GSH-Px), the detection of these antioxidant substances is not universally available for patients with AP due to their relatively higher medical costs. In contrast, biochemical parameters such as serum bilirubin, uric acid, creatinine, and albumin can be easily detected from blood samples at low cost and are among the routine test items in clinical practice. Therefore, these serum parameters might be suitable biomarkers for the detection of antioxidant status in patients with AP. Our results showed that serum TBIL and albumin exhibited not only good diagnostic accuracy for the prediction of SAP but also high predictive capability for in-hospital mortality in patients with AP in two ICU databases.

Recently, a series of diagnostic and prognostic markers alone and in combination have been evaluated in patients with AP^35^-^37^. Although numerous available biomarkers have been proposed for the prediction of prognosis, there remains a lack of specific biomarkers for the early and reliable prediction of AP severity. Hence, our study aimed to identify diagnostic and survival biomarkers for AP from the perspective of serum endogenous antioxidant indexes. While we attempted to include all available variables (serum TBIL, DBIL, indirect bilirubin [IBIL], uric acid, albumin, and creatinine) obtained from the database in our analyses, we excluded serum IBIL and uric acid due to significant missing data. After the multivariate regression analyses, only lower levels of serum albumin and TBIL were independent risk factors for the prediction of SAP and prognosis in patients with AP.

Most studies investigating predictive markers for AP severity or prognosis were limited by relatively small sample sizes^38^-^40^. Hence, the present retrospective study analyzed data from the MIMIC-III, an open-access ICU database of 53,423 distinct admitted adult patients. Furthermore, we also replicated our analyses using another large-scale ICU database (eICU-CRD) to demonstrate the reliability of our findings. Therefore, to our knowledge, this is the first clinical study with the largest sample size to simultaneously analyze the MIMIC-III and eICU-CRD databases to explore the diagnostic and prognostic significance of serum TBIL and albumin in 1,185 cases of patients with AP. Strictly speaking, this clinical research could be considered a multi-center cohort study. The results of this clinical study may provide an important reference to guide clinical practice.

This retrospective analysis with a large sample size explored the diagnostic and prognostic significance of endogenous antioxidant indexes from the MIMIC-III database and validated the results in the eICU-CRD. However, this study has some inevitable limitations. First, although we tried to investigate the diagnostic and prognostic role of endogenous antioxidant indexes, we had to exclude indirect bilirubin (IBIL) and uric acid from our analysis due to significant missing data. Next, both the MIMIC-III and eICU-CRD databases lacked computed tomography (CT) data, which play an important role in the diagnosis of SAP. Finally, our findings lacked power due to the retrospective study design.

## Conclusions

The results of our study showed the prognostic values of both serum TBIL and albumin in patients with AP. The nomograms including serum TBIL and albumin showed good discrimination and calibration abilities in predicting SAP and in-hospital mortality. Further prospective studies with larger sample sizes are needed to confirm the diagnostic and prognostic values of endogenous antioxidant indexes in patients with AP.

## Figures and Tables

**Table 1:** Characteristics of the acute pancreatitis patients on admission

Characteristic	Test set (n=708)			Validation set (n=477)		
	TBIL≥ 1.1 (n=552)	TBIL< 1.1 (n=156)	P value	TBIL≥ 1.1 (n=390)	TBIL< 1.1 (n=87)	P value
Age, year	58.3±16.7	58.4±15.9	0.979	52.4±16.4	56.9±16.6	0.022
Sex, male, n (%)	314 (56.9)	88 (56.4)	0.898	236 (60.5)	52 (59.8)	0.898
Ethnicity, n (%)			0.864			0.716
White	392 (71.0)	108 (69.2)		334 (85.6)	73 (83.9)	
Black	41 (7.4)	15 (9.6)		25 (6.4)	7 (8.0)	
Others	119 (21.6)	33 (21.2)		31 (7.9)	7 (8.0)	
Body mass index, kg/m2	29.7±7.6	30.5±8.7	0.782	30.8±13.9	29.0±8.0	0.494
Comorbidities, n (%)						
Chronic kidney disease	60 (10.9)	23 (14.7)	0.184	30 (7.7)	12 (13.8)	0.069
Hypertension	287 (52.0)	83 (53.2)	0.805	164 (42.1)	45 (51.7)	0.101
Diabetes	131 (23.7)	46 (29.5)	0.150	83 (21.3)	19 (21.8)	0.909
Chronic heart failure	107 (19.4)	38 (24.3)	0.174	24 (6.2)	8 (9.2)	0.305
Complications, n (%)						
Acute kidney injury	306 (55.4)	119 (76.3)	<0.001	73 (18.7)	45 (51.7)	<0.001
Sepsis	161 (29.2)	37 (23.70	<0.001	145 (37.2)	67 (77.0)	<0.001
Therapeutic management, n (%)						
Vasopressor usage	161 (29.2)	87 (55.8)	<0.001	48 (12.3)	34 (39.1)	<0.001
Mechanical ventilation	257 (46.6)	125 (80.1)	<0.001	31 (7.4)	11 (14.9)	0.162
Renal replacement therapy	48 (8.7)	38 (24.4)	<0.001	10 (2.6)	20 (23.0)	<0.001
Clinical management, n (%)						
Endoscopic necrectomy	5 (0.9)	0 (0.0)	0.426	1 (0.3)	2 (2.3)	0.215
Surgical necrectomy	8 (1.4)	1 (0.6)	0.233	6 (1.5)	3 (3.4)	0.237
ERCP	157 (28.4)	20 (12.8)	<0.001	24 (6.2)	4 (4.6)	0.578
Severity of illness, points						
SOFA score	4.8±1.6	7.0±3.6	<0.001	3.6±1.6	6.1±2.9	<0.001
OASIS score	31.8±9.0	37.6±8.7	<0.001	21.1±9.3	29.5±10.3	<0.001
Biochemical data						
MAP, mmHg	65.7±16.4	63.4±12.1	0.071	74.1±29.4	50.9±24.2	<0.001
Leucocyte,×109/L	13.7±7.4	15.0±8.7	0.113	11.6±6.4	12.8±7.0	0.125
Hemoglobin, g/dL	11.0±2.2	10.0±2.0	<0.001	11.7±2.6	9.7±1.9	<0.001
Platelet, ×109/L	270.9±80.4	264.9±63.2	0.708	212.0±81.1	208.9±80.0	0.817
Serum albumin, g/dL	3.0±0.7	2.7±0.7	<0.001	2.9±0.7	2.5±0.7	<0.001
TBIL, mg/dL	3.5±1.2	0.3±0.2	<0.001	3.2±1.2	0.5±0.3	<0.001
DBIL, mg/dL	3.1±1.2	0.2±0.1	<0.001	6.0±2.1	0.5±0.3	<0.001
Lactate, mmol/L	2.3±1.6	2.1±1.0	0.399	2.2±1.1	2.3±1.0	0.068
Lipase, IU/L	139.0 (50.0, 562.0)	140.0 (52.5, 305.5)	0.175	294.0 (97.0, 1175.0)	242.0 (98.5, 854.0)	0.186
Amylase, IU/L	133.0 (60.0, 300.0)	139.0 (40.3, 419.3)	0.280	134.0 (58.0, 428.5)	134.0 (57.5, 370.0)	0.911
Urea nitrogen, mg/dL	25.7±11.0	38.1±17.8	<0.001	19.0±9.7	38.6±14.4	<0.001
Serum creatinine, mg/dL	1.3±0.3	2.2±0.7	<0.001	1.3±0.6	2.9±1.7	<0.001
Alkaline phosphatase, IU/L	168.5±91.2	131.3±30.9	0.031	127.9±80.2	131.8±70.9	0.757
Severe AP, n (%)	94 (17.0)	112 (71.8)	<0.001	39 (10.0)	77 (88.5)	<0.001
Hospital LOS, days	14.8 (7.0, 21.7)	16.0 (10.8, 29.6)	0.199	10.5 (5.5, 19.9)	12.5 (6.3, 21.4)	0.268
In-hospital mortality, (%)	46 (8.3)	45 (28.8)	<0.001	12 (3.1)	17 (19.5)	<0.001

TBIL, total bilirubin; ERCP, endoscopic retrograde cholangiopancreatography; SOFA, sequential organ failure assessment, OASIS, oxford acute severity of illness score; APACHEIV, acute Physiology and Chronic Health Evaluation IV; MAP, mean arterial pressure; DBIL, direct bilirubin; AP, acute pancreatitis; ICU, intensive care unit; LOS, length of stay.

**Table 2:** Logistical analyses for severe acute pancreatitis in the MIMIC-III database

Variables	Unadjusted OR (95%CI)	P value	Adjusted OR (95%CI)	P value
Age, year	0.996 (0.987-1.006)	0.458		
Sex, male, n (%)	1.058 (0.763-1.469)	0.734		
Ethnicity, n (%)				
White	0.876 (0.592-1.298)	0.511		
Black	0.722 (0.360-1.477)	0.359		
Others	Ref.	-		
Body mass index, kg/m^2^	1.021 (0.999-1.047)	0.057	1.004 (0.956-1.054)	0.876
Comorbidities, n (%)				
Chronic kidney disease	2.297 (1.441-3.663)	<0.001	2.596 (0.884-4.628)	0.083
Hypertension	0.880 (0.636-1.218)	0.441		
Diabetes	1.218 (0.843-1.759)	0.294		
Chronic heart failure	1.428 (0.969-2.105)	0.072	1.649 (0.685-3.969)	0.264
Severity of illness, points				
SOFA score	1.440 (1.354-1.531)	<0.001	1.326 (1.176-1.495)	<0.001
OASIS score	1.120 (1.096-1.145)	<0.001	1.054 (0.943-1.178)	0.350
Biochemical data				
MAP, mmHg	0.968 (0.957-0.979)	<0.001	0.998 (0.976-1.022)	0.896
Leucocyte,×10^9^/L	1.005 (0.987-1.024)	0.584		
Hemoglobin, g/dL	0.764 (0.700-0.835)	<0.001	0.706 (0.578-0.863)	0.001
Platelet, ×10^9^/L	0.998 (0.996-0.999)	0.014	1.000 (0.977-1.022)	0.978
Serum albumin, g/L	0.441 (0.340-0.571)	<0.001	0.890 (0.819-0.967)	0.006
Total bilirubin, mg/dL	0.651 (0.571-0.742)	<0.001	0.740 (0.616-0.889)	0.001
Direct bilirubin, mg/dL	1.059 (1.001-1.121)	0.048	1.050 (0.931-1.185)	0.422
Lactate, mmol/L	1.063 (0.978-1.156)	0.152		
Lipase, IU/L	1.000 (0.998-1.002)	0.198		
Amylase, IU/L	1.001 (0.999-1.002)	0.404		
Urea nitrogen, mg/dL	1.039 (1.030-1.047)	<0.001	1.030 (1.015-1.045)	<0.001
Serum creatinine, mg/dL	1.710 (1.492-1.959)	<0.001	1.422 (0.905-2.235)	0.127
Alkaline phosphatase, IU/L	1.000 (0.998-1.001)	0.982		

OR, odds ratio; 95%CI, 95% confidence interval; SOFA, sequential organ failure assessment, OASIS, oxford acute severity of illness score; MAP, mean arterial pressure.

**Table 3:** Univariate and multivariate Cox regression for prognostic factors in patients with AP in MIMIC-III database

	Unadjusted HR (95%CI)	P	Adjusted HR (95%CI)	P
Age, year	1.034 (1.020-1.049)	<0.001	1.047 (1.024-1.071)	<0.001
Sex, male, n (%)	1.061 (0.693-1.625)	0.784		
Ethnicity, n (%)				
White	0.448 (0.289-0.695)	<0.001	0.358 (0.185-1.069)	0.092
Black	0.347 (0.134-0.899)	0.029	0.374 (0.100-1.283)	0.115
Others	Ref.	-	-	-
BMI, kg/m2	1.000 (0.972-1.030)	0.985		
Comorbidities, n (%)				
Chronic kidney disease	1.080 (0.608-1.919)	0.792		
Hypertension	1.239 (0.815-1.885)	0.315		
Diabetes	1.366 (0.869-2.149)	0.176		
Chronic heart failure	1.863 (1.197-2.866)	0.006	1.874 (0.945-3.719)	0.072
Complication, n (%)				
Acute kidney injury	1.906 (1.087-3.342)	0.024	1.197 (0.517-2.773)	0.674
Sepsis	2.254 (1.435-3.541)	<0.001	1.648 (0.847-3.207)	0.141
Therapeutic management, n (%)				
Vasopressor usage	3.017 (1.848-4.927)	<0.001	1.791 (0.767-3.181)	0.178
Mechanical ventilation	1.539 (0.920-2.574)	0.101		
Renal replacement therapy	1.817 (1.156-2.855)	0.010	1.493 (0.718-3.105)	0.283
Clinical management, n (%)				
Endoscopic necrectomy	0.433 (0.004-28.457)	0.344		
Surgical necrectomy	1.143 (0.356-3.665)	0.823		
ERCP	0.813 (0.485-1.365)	0.434		
Severity of illness, points				
SOFA score	1.140 (1.085-1.198)	<0.001	1.222 (1.110-1.346)	<0.001
OASIS score	1.049 (1.026-1.071)	<0.001	0.961 (0.919-1.006)	0.086
Biochemical data				
MAP, mmHg	0.986 (0.973-0.999)	0.038	1.004 (0.984-1.026)	0.675
Leucocyte,×10^9^/L	1.022 (1.008-1.037)	0.003	1.035 (1.013-1.058)	0.002
Hemoglobin, g/dL	1.038 (0.931-1.158)	0.502		
Platelet, ×10^9^/L	0.972 (0.584-1.144)	0.240		
Serum albumin, g/L	0.916 (0.905-0.968)	<0.001	0.962 (0.927-0.998)	0.037
Total bilirubin, mg/dL	0.761 (0.651-0.890)	0.001	0.768 (0.635-0.928)	0.006
Direct bilirubin, mg/dL	1.091 (1.049-1.134)	<0.001	1.002 (0.966-1.038)	0.927
Lactate, mmol/L	1.134 (1.073-1.198)	<0.001	1.075 (0.985-1.173)	0.104
Lipase, IU/L	1.000 (0.998-1.001)	0.174		
Amylase, IU/L	1.001 (0.999-1.002)	0.950		
Urea nitrogen, mg/dL	1.014 (1.007-1.020)	<0.001	1.001 (0.989-1.012)	0.887
Serum creatinine, mg/dL	1.144 (1.033-1.267)	<0.001	0.972 (0.738-1.281)	0.842
Alkaline phosphatase, IU/L	1.002 (0.998-1.002)	0.751		
SAP	1.878 (1.235-2.856)	0.003	1.097 (0.496-2.428)	0.819

HR, hazard ratio; 95%CI, 95% confidence interval; ERCP, edoscopic retrograde cholangiopancreatography; SOFA, sequential organ failure assessment, OASIS, oxford acute severity of illness score; MAP, mean arterial pressure; DBIL, direct bilirubin; SAP, severe acute pancreatitis.

**Table 4:** Comparison of predictive performances of the nomogram, SOFA and OASIS in the MIMIC-III and e-ICU databases

Database		Nomogram (C-index: 95%CI)	SOFA (C-index: 95%CI)	OASIS (C-index: 95%CI)	P_ns_ value	P_no_ value
MIMIC-III	SAP	0.855(0.824-0.885)	0.818(0.784-0.852)	0.758(0.719-0.798)	0.001	<0.001
	Mortality	0.821(0.771-0.872)	0.760 (0.706-0.803)	0.729 (0.675-0.813)	0.001	0.0168
e-ICU	SAP	0.879(0.844-0.914)	0.801 (0.756-0.846)	0.746 (0.695-0.798)	<0.001	<0.001
	Mortality	0.822(0.744-0.901)	0.752 (0.673-0.831)	0.741(0.670-0.812)	0.0029	0.0336

P_ns_ refers to the comparison between the nomogram and SOFA. P_no_ refers to the comparison between the nomogram and OASIS.

## Abbreviations

AP: acute pancreatitis
TBIL: total bilirubin
ERCP: endoscopic retrograde cholangiopancreatography
SOFA: sequential organ failure assessment
OASIS: oxford acute severity of illness score
APACHEIV: Acute Physiology and Chronic Health Evaluation IV
MAP: mean arterial pressure
DBIL: direct bilirubin
AP: acute pancreatitis
ICU: intensive care unit
LOS: length of stay
HR: hazard ratio
95%CI: 95% confidence interval
